# Delivering and beyond: insights about the ExpandTPT community advisory board experience in Brazil

**DOI:** 10.7189/jogh.16.03026

**Published:** 2026-08-28

**Authors:** Ezio Távora dos Santos Filho, Anete Trajman

**Affiliations:** 1 Rede Brasileira de Pesquisas em Tuberculose, Rio de Janeiro, Brazil; 2 Medical School, Universidade Federal do Rio de Janeiro, Rio de Janeiro, Brazil; 3 McGill International Tuberculosis Centre, McGill University, Montreal, Canada

**Keywords:** tuberculosis, prevention, tuberculosis preventive treatment, civil society engagement, community advisory board

## Abstract

Expansion of tuberculosis (TB) preventive treatment (TPT) in contacts has been a challenge worldwide. Brazil is no exception, despite the publication of TB treatment guidelines as early as 1980. The ExpandTPT operational research programme was conducted from 2023 to 2025 in two phases across eight Brazilian high TB-burden cities to scale up TPT among contacts as per national guidelines. The programme was a partnership among the Brazilian TB Research Network (REDE-TB), the national TB programme, and civil society, consisting mainly of capacity building and local service reorganisation based on needs assessment at the municipal and clinic level. During the ExpandTPT implementation, TPT prescriptions increased by 70% in the five participating cities, which is a 30% increase in overall national TPT prescriptions from 2023 to 2024. In this viewpoint, we describe the working method of the ExpandTPT community advisory board, its key elements, role in the programme, and the authors’ insights into its challenges and contribution to community engagement in TB research. Lessons learned from this experience may contribute to other operational research projects in public health.

Tuberculosis (TB) preventive treatment (TPT) is the primary healthcare intervention for reducing the incidence of the disease [1]. However, expansion of TPT coverage worldwide has been a challenge, especially in contacts. The United Nations high-level meeting established a global goal to provide TPT to 30 million close contacts of people with tuberculosis and to 15 million people living with HIV/AIDS (PLHA) in 2023 [2]. The World Health Organization (WHO) estimates that 5.3 million people were provided with TPT in 2024, of which 3.5 million were contacts, representing 25% coverage of the estimated eligible contacts. By contrast, among PLHA newly enrolled in antiretroviral treatment, TPT coverage was 58% [1]. Sub-financing of national TB programmes (NTP) contributes to this scenario, but other barriers still exist [3].

The ExpandTPT study [4] was an operational programme led by the Brazilian TB Research Network (REDE-TB) in partnership with the Brazilian NTP and civil society (CS) advocates, through the establishment of a community advisory board (CAB). The initiative was first funded by the Stop TB partnership (phase 1, in 5 high TB-burden cities) [4] and subsequently by the Brazilian Ministry of Health (phase 2, in 3 additional high TB-burden cities) located across 4 of the 5 Brazilian macro-regions. Here, we report our insights as CAB’s coordinator (ETSF) and the study’s principal investigator (AT) on the experiences of the CAB in the ExpandTPT programme, including its constitution, operating mode, challenges, contributions, and legacy.

## Key term definitions

The term ‘CS representatives’ refers to actors of the social movement on TB, which are community-based CS organisations or networks of people affected by the diseases. They represent people affected by TB and/or HIV/AIDS, populations particularly vulnerable to these diseases, and those working with them. Since in Brazil both TB and antiretroviral medication are offered in the public sector by the *Sistema Único de Saúde* (SUS; Brazilian Unified Public Health System), these individuals are also ‘service users’ [5]. All ExpandTPT CAB members fit at least into one of these concepts. Hence, these terms, and the term ‘activist’, are interchangeable in the context of the present discussion. We avoided the term ‘patient’, but used it here as this word denotes tokenism by health professionals. The words ‘project’ and ‘programme’ refer to ExpandTPT, since the project was designed to be implemented as an operational research programme.

## THE EXPANDTPT PROJECT/PROGRAMME

ExpandTPT was an initiative to provide technical support to selected Brazilian NTPs’ priority municipal TB programmes to properly perform the existing guidelines on contact management at primary care level. This meant training managers and healthcare workers (HCW), including community healthcare workers (CHW), to identify and reach out to contacts, screen, treat and prevent TB. Brazil has been recommending TPT for contacts under 16 years of age since the 1980s if they test positive for TB infection and do not have symptoms or radiological signs of TB, as well as for contacts of any age, since 2011 [6]. However, until today, very few contacts are assessed and treated [7].

Therefore, the project was designed as an implementation programme in close partnership with the NTP, to be performed with diverse activities within a period of 18 months in phases 1 and 2 in 5 and 3 cities, respectively. For each phase, a 3-month preparation, a 3-month evaluation (needs assessment) and a 12-month implementation period were planned. The project aimed to empower the pilot municipality managers and services to autonomously further expand TPT scaling up.

ExpandTPT methods were based on the ACT4 cluster randomised clinical trial, which identified barriers and implemented solutions for TPT expansion in contacts in Brazil [8] and 5 additional countries [9]. In the ACT4 trial, obstacles were assessed through questionnaires to persons with TB, their contacts, HCW and CHW. However, understanding how healthcare services are structured and the internal dynamics among HCW and their leadership (unit managers, programme managers) at primary care level was not achieved.

In ExpandTPT, obstacles to access or comply to TPT were identified by means of a needs assessment questionnaire to participating clinics [10], and through technical visits. The visits involved the local TB managers, all TB service managers, representatives of the Brazilian NTP, ExpandTPT research team and its CAB members.

Solutions were decided in agreement during the technical visits and subsequent interactions among all involved parties. Capacity building – virtual and in-service – was a common solution in all cities. During capacity-building virtual sessions, local CAB members provided feedback to the TB trainers. Training was provided separately to HCW and CHW.

The CAB brought additional information for understanding barriers and finding solutions, and most CAB members are often close to the CHW who are key informants, who are the ones who reach out to people failing to present to the clinics or discontinuing assessment and treatment, and who know where and in which conditions contacts live.

The primary outcome of the study was the proportion of TPT initiated per 100 TPT-eligible contacts, considering TB prevalence, average number of contacts expected per index patient and proportion of contacts with TB infection as per national guidelines [6], in the cities of implementation and the control cities (all other capitals in the country), compared in an interrupted time series model. The source of data was the national information system. In phase 1, during the 12-month intervention period from October 2023 to September 2024, there was a 1.5% (95% confidence interval = 0.1–2.9, *P* = 0.037) higher increase of the primary outcome in the intervention cities than in the remaining control capitals (data not published). This corresponded to a 70% increase in the absolute number of TPT in 5 cities [4], which is a 30% increase in overall TPT notifications in the country in 2024 [7].

### CAB’s concept

As per the general concept, a CAB is an advisory committee, composed of community members, expected to perform mainly an advisory role in a study or programme. It may be created for a specific aim, to follow up and provide input to a study or programme (as in the current case) or be created with a general aim to follow up national or international research policies (*e.g.* the Brazilian National TB CAB, or the Global TB CAB). In both cases, the common goal is to bring community actors into the policy decision process and influence the incorporation of new technologies with the contribution by those most interested.

Conceiving a study-specific CAB aims to enhance its contribution through proper interaction with stakeholders and the study team. In this case, CAB members are supposed to contribute from the inception of the plan, and follow up throughout the study’s development, until the dissemination of its results and implementation of policies according to the study’s findings [11]. The capacity of a CAB to influence policy makers and policy implementers is the most significant aspect of a CAB to be evaluated. In the Brazilian context, CS participation in official Brazilian health fora facilitates CAB’s impact (Text S1 in the **Online Supplementary Document**). To most studies or programmes, however, a CAB functions as an external element, a follow-up advisory committee.

### Understanding CAB members’ identity and role

TB advocates are mostly people affected or vulnerable to the disease, members of community-based organisations and their networks. They relate closely to health programmes and health units due to their activism, and hold particular knowledge about access to health services, as they identify themselves as public health ‘service users’.

In recent years, there has been a slow but growing understanding and recognition by the WHO that these community advocates are key stakeholders for the improvement of health policies and should be engaged in research and in health policy-making [12,13], due to the understanding that CS representatives bring legitimacy, establish demand for goods and services and help accelerate the policy change process. This participation takes place in the complex environment of policy and decision-making, where the power dynamics often exclude the individuals who are the most interested subjects – the users of health services. Following the HIV/AIDS large experience on engaging communities in clinical, operational and implementation studies, TB has increasingly adopted this pattern [5].

Yet, this process of community engagement in research is not straightforward or uniform; it varies according to the scenarios and composition. Moreover, it has many limitations and faces serious obstacles, the most common being: (a) lack of formal expertise or professional skills that would allow advocates’ input and feedback to be taken seriously; (b) lack of resources that could support their activities on a regular basis, which could increase their credibility; (c) prejudice and discrimination towards people with lesser conditions and education.

The role of CS advocates/representatives in a technical health environment – a health unit or a health programme – is not clearly established, nor expected, even considering their role in health councils or committees. There is not a ‘reserved’ or ‘expected’ place for advocates to participate, providing technical or methodological input, suggestions, recommendation in the local-level programmes or services. First, because they are not part of the official structure, as they are not public servants, nor hired as consultants nor as service providers. Second, due to their wide range of educational levels and different professional backgrounds. Finally, their input may be seen with suspicion (fear of monitoring or criticism to the higher levels) [5], and is often not understood due to a lack of formal mechanisms, or seen as inconvenient, inappropriate, external, or not a professional opinion to be considered. The clashes between two perspectives increase the limitations for cooperation (Text S1 in the **Online Supplementary Document**).

### The ExpandTPT CAB experience

The ExpandTPT project planned the participation of a specific study CAB from its early stages. The coordinator (ETSF) was indicated by the principal investigator (AT), based on his extensive previous experience. The coordinator was responsible for establishing selection criteria and selecting members. The selection process (Texts S2 and S3 in the **Online Supplementary Document**) started with mapping potential candidates among TB activists in the respective cities, using the snowball method to reach further interested people. During interviews with the coordinator, gender balance and equity in terms of education and social vulnerability were sought. Criteria for selection included experience in participating in health councils, committees, networks and other CS fora. All members signed the confidentiality agreement and the terms of reference (Texts S3–5 in the **Online Supplementary Document**).

CAB members participated in monthly CAB meetings, ExpandTPT educational activities, preparation of the educational material to CHW and dissemination activities where they advocated for policy change. Given the CAB was funded within the project structure, readers may question its independence and autonomy. Clarifying mechanisms that protected the CAB’s advisory integrity and avoided tokenism increased trust and methodological robustness (Texts S6 and S7 in the **Online Supplementary Document**).

The achievements by the ExpandTPT CAB could be synthesised in two aspects: solid interplay of researchers, NTP and civil society, and direct contribution to policy change (Text S8 in the **Online Supplementary Document**). Although not binding, as buy-in by managers and policy decision-makers is not guaranteed, the direct recommendations by CAB members to TB programme managers and health authorities produced an effect in the power dynamics of TB policies which was reported by TB managers themselves (Text S9 in the **Online Supplementary Document**).

The decision-making and policy definition process must consider the context, actors and dynamics in place. In this sense, two examples of the direct contribution by the ExpandTPT CAB to this process are to be highlighted: change in the national guideline on HCW training for TST application and reading; and shifting NTP’s approach from surveillance to primary care logic (Texts S8 and S10 in the **Online Supplementary Document**).

### Main challenges and solutions of the ExpandTPT CAB

CAB members, in most studies or projects where they perform the community engagement component, are ‘often not seen as participants of the project team’, but as aside, external subjects. This was no exception in ExpandTPT, whose local managers were initially suspicious of and resistant to community participation. Eventually, considerable recognition by TB managers and health professionals at the three levels (federal, state and municipal) was achieved, with some examples of partnership. Yet, closer collaboration was not uniform, and strong reservations remained at some other local programmes, as would be predictable in a biomedical-centred logic environment, where ‘patients’ do not have a role to play.

CAB members’ performance in different programmes’ activities (*e.g.* training) varied given the heterogeneity of educational levels and personalities across cities. Individual skills were one of the determinants of their performance in the ExpandTPT activities; other determinants remained mostly in the power dynamics with other key actors, namely programme or units’ managers and HCW, and their buy-in of community participation; and local policy priorities carried out by the municipal health secretariats and TB programmes.

Not all city managers understood the potential role of communities in the programme’s aim towards the expansion of TPT. Therefore, some missed opportunities were noted. As an example, some community-led capacity building for CHWs led by CAB members, which took place in some cities at the beginning of the intervention phase, generated further training demand by different health services. Yet, the local programmes did not authorise further activities beyond the originally planned units. The justification was the lack of availability of local programmes’ officials to lead the initiative, discouraging autonomy by community members. This ended by interrupting a natural demand that could have further stimulated TPT expansion in the respective cities. On the other hand, cities like Manaus multiplied CAB members’ activities and extended them to riverside municipalities in the Amazonas state. Another significant barrier was the lack of interest by managers and HCW during the in-person training sessions, who were not used to community participation in technical training or any community-led activity in their domain. Nevertheless, overall feedback regarding CS participation was positive in all cities. Retention of members throughout the whole duration of the project was an additional challenge. Motivation was achieved by their learning experience, active participation and fair compensation for their time (data not published). Budgeting for adequate financial compensation for CAB members is a sensitive and relevant aspect to ensure participation and members' performance (Text S11 in the **Online Supplementary Document**).

ExpandTPT CAB’s experience allowed its members, study team, and health managers to learn and accumulate knowledge transferable to other CABs (Text S12 in the **Online Supplementary Document**).

Finally, sustainability of the achievements is a special concern. Recent NTP statistics of TPT indicators in 2025 raise concern about the sustainability of the progress made during the project. [7] Current efforts by the CAB member include ensuring that municipal managers of participating cities sustain the activities and that national managers scale up to other cities.

## CONCLUSIONS

With a variety of profiles and skills, from sex workers to college professors, the ExpandTPT CAB members developed activities and interacted directly with researchers, trainers, HCW, managers and health authorities, as legitimate representatives of vulnerable populations and people affected. They had the opportunity to voice themselves and proved to understand the limitations of the services to expand TPT and, most importantly, provided direct recommendations to improve and sustain this effort.

Ranging from knowledge about TB infection and prevention, training professionals, tracing contacts, network of care and intersectoral articulation, organisational commitment and flow of information within the health units, CAB members went far beyond just production and dissemination of educational materials and funding discussions.

Considering all limitations of time and resources, the authors understand that ExpandTPT CAB consisted of a robust, multifaceted and plural response to the complex and diverse needs of the study and programme in a continental country. Achievements were based on concerted work by experienced professionals committed to trust and confidence among them, to common understanding, and to individual effort by many people, from ExpandTPT CAB members to the study team, the municipal actors and the NTP team.

In our view, this CAB confirmed the benefits of community engagement in policy implementation and the results of a planned, supported and funded initiative, meeting the needs of national commitments towards elimination of TB as a public health problem.

## Additional material


Online Supplementary Document
