# Supplementary material for: Delivering and beyond: insights about the ExpandTPT community advisory board experience in Brazil
Source: J Glob Health. 2026 Aug 28;16:03026. doi: 10.7189/jogh.16.03026 (PMC13521486; doi:10.7189/jogh.16.03026)
Supplement: Supplementary file 1 [file jogh-16-03026-s001.pdf]

Supplement to: Távora dos Santos-Filho E, Trajman A. Delivering and beyond: insights about the ExpandTPT community advisory board experience in Brazil. J Glob Health. 2026;16:03026.

Supplement material

Text S1: Civil society (CS) participation in Brazilian health fora

The clashing perspective from those who feel entitled to provide feedback into the services and programmes (CS members) and those who understand they are unqualified to do so (HCW and program managers) must be understood within the complex health policy framework scenario in Brazil. Affected people representatives have a constitutionally previewed role in the country's health policy's structure: they compose the "users' segment" – the health service users – in the 4 levels (district, municipal, state, federal) of the Councils of Health. The councils of health are composed equally by three "segments": "users" (50%), "workers" (healthcare workers, 25%), and "managers" (health or program managers, 25%). They discuss, define and recommend the health policies to be implemented by the Executive Power (Secretaries or Ministry of Health). This structure is in place since the creation of the SUS by the Brazilian Federal Constitution of 1988, regulated by Law No. 8,080 in 1990. The 1988 Constitution established health as a right of all, and its provision a states' duty, therefore universal and free access to health for all Brazilians and foreign residents. Free and universal meaning non-contributively, with full costs – from lowest to highest complexity – covered entirely by the Public Treasures of the three levels of the State.

Therefore, there is a legitim and legal role to the CS representatives in the structure of the Brazilian Health System. Although not systematically or directly funded, the CS organizations, community-based in particular, are settled to provide a peer-to-peer support, grassroots response on local level, and networking to guarantee access to services and programmes. They actively participate in the different councils, committees and policy fora. Yet the knot of the problem is that any guideline/recommendation should be "officially" provided from the higher level of the health structure, and not directly to health units or services.

Thus, as far as TPT expansion is concerned, given the services and programmes have not performed well along decades of preventive policies despite clear recommendations, it seems to be key to introducing in the scenario new subjects with a different knowledge and expectations.

Text S2: The CAB coordinator previous experience

The CAB coordinator (ETSF) was invited by the PI (AT) based on his extensive experience in leading CAB. ETSF had previously coordinated several TB CAB in Brazil, for different studies, such as PROVE-IT (USAID's TREAT-TB Grant), SimpliciTB (TB Alliance); MIRA-TB (Brazilian MoH funded), M-10, Impac19TB (Brazilian MoH funded). He also participated in international CAB such as CRAG for the U.S.' CDC, the Global TB CAB (run by the Treatment Action Group, NY), the EU-PEARL (European Union) and the Reflate Study Scientific Meeting

for the French Agency for HIV/AIDS Research, ANRS France. He was also responsible for creating and running CAB in Mongolia, Moldova, Georgia and India for the STREAM (the Union/Vital Strategies) study since 2015, and coordinated the CE for all 13 sites, in 7 countries, also including Ethiopia, South Africa and Uganda of all sites in 2021.

Text S3: Member selection criteria and process

The ***common criterion*** to all members was the understanding or at least the **interest in learning TPT as a key strategy** in the fight against TB. Since influencing policy-making process for policy change is a general aim of most CAB, another common background was to have some ***experience participating in health councils, committees, networks and forums***. This was also a criterion to influence the implementation of the project. Moreover, their familiarity or connection with local program managers and services would be an asset for their participation in the CAB.

A ***direct selective process*** was carried out by the coordinator, who mapped suitable or potentially interested members in the cities where the program was to be established. This was possible due to the existing network of TB and other SC advocates. Potential members were indicated by means of the "snowball" method. The coordinator conducted online interviews with each candidate separately; those interested and available received a copy of the term of reference (see Supplement 3) and adhered electronically to the membership. The same process was applied in phases 1 and 2 of the program, and whenever a member had to be replaced.

Three CAB members were selected for each site, meant to secure participation of at least one member in meetings and activities when there were agenda conflicts. In some cities, like Porto Alegre, Rio de Janeiro, and São Paulo, just two CAB members remained for most of the program, but the practice indicated that having three enhances the chance to guarantee participation in training and activities. Despite some replacements, the CAB worked with 15 members most of the time, out of 22 members that participated at some point.

The coordination observed ***gender balance*** – the total composition of members were 11 women to 11 men – and ***equity***, in terms of education and representativeness of minorities who may self-identify as vulnerable to TB, such as female sex workers (n=2).

Text S4: ExpandTPT CAB composition

As described in Supplement 2, level education was not a selection criterion for inclusion in the ExpandTPT CAB. As a result, the composition of the ExpandTPT CAB ranged from university teachers to elementary school level of instruction members. It included retired or acting health

managers or professionals, grass-root community advocates from different TB networks, such as sex-workers and representatives of the LGBT+ organizations.

The heterogeneity of professional and educational background did not represent a major challenge, since they had all participated in councils or committees which are marked by non-uniformity of profiles.

The level of education of the 22 ExpandTPT CAB members who participated at some point were: elementary or basic education (n=3); high school (intermediary) education (n=5); university level (undergraduate, n=9), Master's degree (n=3), and Doctorate degree (n=1) or in a Doctoral program (n=1).

Text S5: Terms of Reference (ToR)

Key aspects of the ToR signed by CAB members are listed below:

Requirements: commitment with TB advocacy, and interest in understanding and help expanding TPT; availability to follow up the study/program implementation in a flexible manner for a few hours weekly; no educational background was required.

Conditions: working on a volunteer basis, with a small monthly support to cover communication and transportation expenses; flexibility due to unpredictable agenda changes; respect to differences of opinion and perspective; minimum participation at the online and in presential activities; filling up short participation reports/feedback.

Regularity: Online general meetings with all CAB members were held monthly, in average. Separate meetings with members at each city/site were done more frequently due to extensive training and other activities agenda.

Communication: a general WhatsApp group was created with all members and Program Director, and other local groups with respective CAB members with local TB managers and Focal Points. A second WhatsApp group was created exclusively among CAB members at each city, to allow free comments and avoid embarrassment in case of sensitive remarks on the program.

Solution of conflicts: to be carried out collectively, with the ultimate decision by the coordination.

Text S6: Scope, intent and limits of regular ExpandTPT CAB activities

a. **Training** – The ExpandTPT program was run from Rio de Janeiro (REDE-TB, UFRJ) in partnership with the NTP in Brasilia. It delivered initially a series of online and in person training for program or health units' managers and HCW, which included CHW. CAB members participated regularly in the online training and issued electronic participation reports, where they provided input and observational remarks. These reports were summarized by CAB coordinator and reported to the PI and Project Director, resulting in several adjustments in the method and approach applied in the online training. Those remarks regarded engagement of the audience, missing points and tips to enhance participation. Length of training, time for Q&A, language used, content of online questions were the most common comments. Feedback by trainees were very positive.

   CAB members participated in training more actively in those cities where they were included in the program, and when they delivered sessions on community participation. CAB members also provided input and feedback to the coordination and eventually directly to the in-person trainers.

b. **Revision and production of printed and electronic materials** – As a core function by CAB, members were consulted on the validity, format and language of different materials targeted to contacts of index cases, or materials to HCW.

   Judging the language of the ExpandTPT materials for HCW were inaccessible to the CHW, CAB members carried out three rounds of revision of these materials, in direct consultation with 197 CHW (20 in focus groups, 177 in face-to-face interviews). The entire production of the "*Guia d@s ACS para TPT*" (or "TPT Guide for CHW " - https://redetb.org.br/guia-das-acs-para-tpt/) was delivered in 5 months. The guide was widely distributed along two years, and received many acknowledgements, such as by CHW representatives at the São Paulo ExpandTPT Seminar in August 2024. This process is further detailed in Supplement 6.

   ExpandTPT CAB members also used the programs' materials to produce other TPT materials targeted and tailored for specific regional references, like in Manaus and João Pessoa, or to other publics, such as people deprived of liberty, in Porto Alegre.

   Most materials were made available both on electronic and printed versions.

c. **General meetings, webinars and seminars** – Besides the usual ***monthly (internal) meetings*** of the CAB, members actively organized or presented their perception of the programs' development, and feedback from other meetings and program activities (see Supplements 7 & 8).

   Yearly national meetings were held in a webinar format, counting always with the participation of the NTP manager and team members; plus, the local TB programmes'

coordination with their respective Focal Points. A ***national seminar*** was held in Brasília on March 13, 2025 (see Supplement 7).

More than just reporting their perspective of the ExpandTPT program development at each city/site, their presentation at those meetings and webinars provided critical feedback both for the NTP and the local managers (see Supplements 7 & 8).

d. **Conferences** – besides the webinars and the ExpandTPT Seminar in 2025, some ExpandTPT CAB members delivered oral presentation or participated in diverse national and international conferences, such as the REDE-TB's National Workshops at Brazilian Tropical Medicine Conference (MedTrop) in 2023, 2024 and 2025. In 2024, one ExpandTPT CAB member participated in World Conference on Lung Health (the Union Conference) in Bali, Indonesia, sponsored by the NTP-MoH. They have also participated in several other conferences and symposiums at different universities in the country.
e. **Advocacy** – Although participation of members in TB or HIV/AIDS fora, committees and councils were already part of most CAB members' activities, bringing TPT into the agenda of different policy discussion forums was often registered. Moreover, the advocates also articulated specific meetings with Municipal Health Secretaries in cities like Recife and João Pessoa, which were relevant for the advance of the program's expansion.
f. **Expanding initiatives –** Two highlights are worth citing here: In Nova Iguaçu, CAB members enhanced the existing Rio de Janeiro State Health Authority's programs for health and education with TB and TPT sessions and workshops. A Working Group was constituted with the participation of community representatives and that unfolded to a Social Service course at the Iguaçu University. Moreover, two ExpandTPT CAB members published a paper on the CAB experiences in 2025.[1]

   In João Pessoa (Paraíba State), CAB members autonomously designed, submitted and were awarded first place at a national call for TB, HIV projects by the Brazilian Ministry of Health, in partnership with PAHO. The "*Projeto na rua livre da tuberculose*", submitted by NGO CORDEL Vida, in João Pessoa, PB designed an expansion of the program for specific target populations, namely outreach and support to homeless people vulnerable or with TB, and focus on sensitizing CHW to TB prevention and care. This autonomous project has been running in parallel to the ExpandTPT itself, since May 2025, with clear impact in the policies. The greatest achievement was sensitizing and engaging the CHW in João Pessoa, a strong union working category, who were not convinced of the efficacy and conditions to perform TPT in that city. This initiative entirely changed the scenario of collaboration and integration at local primary care level in Paraíba's capital.

Text S7: Production process of TPT training material for CHW

While reviewing the printed materials for HCW, CAB members pointed out that those materials would not be friendly enough for CHW, as they would not be familiar with many medical terms, acronyms and other technicalities. CAB members carried out an initial revision and drafted a more accessible material (1st version). Followed a sequence of two-round consultations with CHW at all five initial sites was carried out: firstly virtual, then in presence, allowing a deeper analysis of the content, format and language. The first round of consultation consisted in a double focus group to revise the 1st draft version produced by the ExpandTPT CAB itself. Each focus group consisted in 10 CHW, 2 from each city, working in different health units. These 2 focus groups were conducted online in a sequence of 2 days by a survey specialist, experienced market researcher and an advocate herself. With the output of the focus group, a 2nd version of the CHW's Guide to TPT was ready. Finally, the second round of direct consultation took place at each city, this time conducted by local ExpandTPT CAB members, who gathered with CHW in two healthcare units located in different parts of their cities, in different days. CHW thoroughly revised the 2nd version. CAB members assembled the comments and editing suggestions (or requirements) by CHW; those were edited by the CAB coordinator and revised by the principal investigator and MoH's advisors. This resulted in a 3rd and final version, which included a friendly glossary of terms. The final print format chosen by the CHW fitted in their backpack. This direct consultation counted 197 CHW (20 in focus groups, 177 in person). A few weeks after printing, another group of roughly 200 HCW (many having taken part in the consultation) in all 5 cities validated the CHW's Guide to TPT (available at https://redetb.org.br/guia-dos-acs-para-tpt/). The whole process took less than five months to be completed, from May to September 2023. Four printing between 2023 and 2025 totalled 14,000 copies.

Text S8: Achievements of the ExpandTPT CAB

The ExpandTPT CAB promoted general National virtual meetings, with the NTP and local TB managers, on 24-Nov-2023, 06-Dec-2024 and 15-Dec-2025. They were key organizers and presenters of the National ExpandTPT Seminar in Brasilia "Evidence and action: The contribution by ExpandTPT for TB elimination" ("*Evidências e Ação: A contribuição do ExpandTPT para a eliminação da tuberculose*"), March 13th, 2025, Brasília, DF.

During the abovementioned presential seminar in Brasilia, the ExpandTPT CAB members from all the eight cities presented a succinct report and their views on the possible ways to sustain the TPT expansion on a long run. In the audience of this seminar were the head of the National

Secretary of Health Surveillance; the NTP coordinator and team, as well as most heads of local and state TB programs.

In late 2025, based on the CAB's perception of the limitations for TPT expansion at primary care level, the ExpandTPT CAB coordinator suggested the NTP to articulate and carry out an integrative session between the Primary Care and the Surveillance Secretaries of the MoH. A National Webinar titled "The TPT expansion in Brazil – sustainable ways" ("*A expansão do tratamento preventivo da tuberculose (TPT) no Brasil – caminhos sustentáveis"*) took place on March 2nd, 2026, on the MoH's webinars' platform, opening the MoH's official calendar allusive to the World TB Day. 1,154 participants attended this event, promoted by the NTP. This was a follow-up discussion on the role of the Primary Care Secretariat on the sustainability and roll out of TB prevention services (see details in Supplement 8). Comments by the NTP manager highlighted the rapid progress and cooperation between the two MoH's secretariats since the organization of the webinar.

Therefore, the most significant achievement were the **direct recommendations provided to local, state and federal programmes by the ExpandTPT CAB**, virtually and in presence, in several regular opportunities from start until the conclusion of the project.

Recommendations provided by ExpandTPT CAB members **indicated where revision and adjustments of program plans** were needed, particularly at local level. Moreover, they presented feasible manners to **increase sustainability of activities, partnerships and funding.** These recommendations aimed to guarantee both municipal TPT expansion and community participation, as the advocates function unfunded at local activities, counting with no regular financial support. These outputs varied from indication of potential local partners to be engaged, and holding local health authorities accountable by means of Health Councils' monitoring, to the use of existing funding channels and other alternatives for the TPT expansion and community funding.

This helped **building a trusting and collaborative atmosphere** among ExpandTPT team with CAB members, NTP and local TB programs. The NTP has acknowledged the ExpandTPT CAB on several occasions for providing direct recommendations and having active participation in the TPT expansion process. Yet, limitation remained in the buy-in by TB program managers, especially at local level.

Text S9: Summary of recommendations by ExpandTPT CAB

Follow 10 priority points raised by ExpandTPT CAB members. These recommendations were addressed to the NTP and municipal TB programs.

- Include in municipal planning the costs of regular training in TPT and civil society participation in TB actions, e.g., facilitating internal workshops and reach out activities.
- Establish regular internal workshops to the health units to hear and learn from HCW and CHW, the contacts of people with TB, their experiences and needs, and adapt services accordingly.
- Guarantee resources for TPT community preparedness and the inclusion of civil society in actions aimed at the most vulnerable populations, such as people experiencing homelessness and those deprived of liberty.
- Guarantee a permanent agenda between community organizations and municipal management for monitoring training and actively seeking out TB contacts.
- Ensure financial and technical resources for civil society participation in the strategic planning and execution of prevention actions.
- Simplify and standardize care protocols in TB prevention to strengthen their use by health professionals and properly inform populations at risk.
- Strengthen regular community participation in the planning and execution of activities with other sectors, like education, justice and social protection, including inter sector forums and permanent spaces for territorial dialogue.
- Ensure robust investments in communication for general awareness (public campaigns) and community targeted activities to reach vulnerable populations in continued educational efforts to generate a culture of prevention and effectively expand TPT.
- Include coverage of basic institutional expenses of NGO in public calls for proposals to ensure the sustainability and quality of community work.
- Promote the wise use of existing funding mechanisms from different secretaries (e.g., education, social assistance) to accelerate the implementation of TPT and partially fund community participation.

Text S10: Direct contributions by the ExpandTPT CAB for policy change

The CAB directly contributed to policy change in two specific occasions. Firstly, for the **revision of national recommendations** (guidelines) on TST training: the former requirement[2] of 100 arms to qualify HCW for TST application and reading has been an explicit obstacle for the expansion of TPT in Brazil.[3] The baseline needs assessment conducted by the ExpandTPT project has shown that most primary clinics had no personnel trained to perform TST.[4] The CAB Coordinator and members questioned, on several public events, e.g. the XI National Workshop of the Brazilian TB Research Network (São Paulo, SP, 22-25-November-2024) and the REDE-TB's Seminar (*Simpósio de Tuberculose de Ribeirão Preto*, SP, 12-August-2024: "*Rumo para 2030: interconexões para a eliminação da tuberculose*") these recommendations. This questioning accelerated ongoing discussions within the NTP, its advisory board and opinion-leader experts, also present in these meetings. Based on the ExpandTPT experience,[4] additional

previous national evidence[5] and a systematic review on current practice (submitted), new recommendations were published in December 2024.[6]

Secondly, ExpandTPT CAB functioned as a catalysing agent while **promoting the collaboration of the key Secretariats of the MoH**. According to Fernanda Dockhorn, NTP Manager, the negotiation between the NTP, which is subordinated to the Secretary of Health Surveillance (*Secretaria de Vigilância em Saúde e Ambiente*, SVSA), and the Secretary of Primary Care Services (*Secretaria de Atenção Primária em Saúde*, SAPS), "the process of negotiation for the webinar contributed to improve communication and accelerate exchange among both Secretaries. This has been progressing since then, and we are advancing planning collaborative activities and will create the path for joint initiatives. (WhatsApp communication between ETSF and Fernanda Dockhorn, NTP coordinator, on 27-February-2026 and 04-May-2026")

According to presenter Dr. Edna Ferreira, R.N, the webinar in March 2026 "contributed to improving the dialogue between surveillance and care, by highlighting tensions and the shared responsibility of these spheres in the articulation of care for people with tuberculosis in the SUS" (WhatsApp communication between ETSF and Edna Ferreira on 04-May-2026).

"For the Secretary of Primary Care Service, the preparation and the webinar itself in March 2026, were excellent opportunities to bring the Secretariats *even closer*, strengthen dialogue, and enable joint actions that will allow for adequate care for people affected by tuberculosis and their contacts, in an effort to *introduce* and *reinforce* the practice of disease prevention" (Statement by Rafaela Alves Marinho, Coordinator of Transmissible Chronic Disease Care at Primary Level (COCT-CGCOC) of the Department for Health Promotion (DEPROS) of SAPS, MoH over WhatsApp with ETSF on 04-May-2026).

Text S11: Budgeting and compensation for members' participation

Properly compensating CAB members for their time, communication and transportation expenses has demonstrated to be another key aspect to be considered while budgeting the study/program. This financial compensation has repercussion in the members' performance throughout its development, as observed in the practice of different studies and occasions. Considering most CAB members are of low or very low income, performing a volunteer-based job, and do not receive salaries from their organizations and networks, their function happens in significant disadvantage, comparable to all the other people engaged, who are either backed by their institutional affiliation, e.g. researchers, managers and health care workers who count with their salaries, or are hired as paid technicians to perform a specific activity. Financial compensation has shown to stimulate participation and engagement of CAB members, besides

functioning as a recognition or acknowledgement by the study/program management of their role.

While budgeting, the requirements by donors or research agencies will help defining the best way to compensate the CAB members. Some agencies do not allow compensation to cover meal expenses, although this is a critical point for the advocates, especially when they are in external activities to their homes or organizations. Transportation and communication are often the best choice, since these are of regularly incurred. Yet financial compensation should be disbursed directly to their accounts, and not otherwise, such as participation in conferences. Participation in conferences and seminars are different forms to stimulate and encourage community participation as they are opportunities to (i) approach and networking with other significant actors; (ii) learn from and exchange with other experiences; (iii) acquire new knowledges; (iv) show off their own performance. But participating in seminars or conferences also represents further work to be done.

Since the ExpandTPT design would require a higher dedication by advocates (timewise), compensation was calculated to reflect CAB participation in virtual or in presence meetings, analysis of documents or research materials. ExpandTPT has provided its CAB members of an amount roughly equivalent to USD $100 monthly (exchange rate by the study beginning) during the intervention phase to each CAB participant, regardless of their educational level or time dedication, since members were supposed to define their participation among them.

By comparison, other study CABs run by the ExpandTPT coordinator offered significantly less (equivalent to USD $20), in Brazilian and other international studies. It must be highlighted most studies do not offer any compensation for the CAB members' time, dedication, and actual expenses (in communication and transportation). This is often seen (and expressed) by CAB members as a form of exploitation and non-recognition by researchers and program managers, reducing power and relevance.

Text S12: The ExpandTPT CAB "Minimum Package" suggestion

According to the parameters used in ExpandTPT CAB, the authors suggest for similar programs the following aspects to be taken into consideration. As per previously referred good participatory guidelines, it is highly recommended study design counts with a CE component and active participation from inception.

CAB Minimum Package

1. **Coordination –** define, select suitable and dedicated coordinator, with good communication and managerial skills and assistant, who will discuss his/her own terms of reference (ToR), including level of effort (LoE) and remuneration. Coordination activity is expected to be of regular and often intense demand and therefore cannot be *ad hoc* or done voluntarily, without remuneration. The coordinator should design the CAB with research team. It is highly recommended to count and budget a CAB coordination assistant and preview financial, administrative support to CAB activities. Depending on CAB's size, this is a requirement, to avoid overload and subsequent delays or even paralysis of activities due managerial issues. Coordination must be proactive and be able to interact openly and regularly with PI and research team. She/he must be included in all research planning and technical discussion. Coordinator has also to have good communication skills to interact with program managers, policy makers, research and regulatory agencies, donors and any other high profile (e.g., media) or low profile (e.g., a person affected). Coordinator must keep control of the team and is accountable for CAB's performance. She/he is responsible for the selection, replacement and maintenance of the CAB.
2. **CAB (or CE) format** – to be defined according to the study/project design (mono or multicentre), considering length, objectives and primary work plan. The CE plan can preview a CAB with its formal, regular activities or other forms of CE, like annual seminars. The primary work plan (draft) is to be further developed with CAB members as a CAB activity.
3. **Budgeting –** should consider, in its planning, the size of CAB or format of any other CE initiative to reach its objectives. This includes regular local activities expenses (transportation, meals, equipment, venue), participation in conferences, or any other activity. Monthly (for CAB members) or ad hoc (for other CE formats) financial support must be secured. The financial flow, availability of funds and financial, administrative support are key; therefore, the supporting organization/institution has to preview the CAB's own organizational demand, which is relevant. Other CAB experiences showed how this item has weakened and discouraged CAB's performance. The proper CAB functioning will depend on correspondent budgeting and good financial performance.
4. **Members selection –** should start with mapping existing organizations and profile of individuals at each site. Selection must previously establish criteria, which required developing and putting forward Terms of Reference, which must contain all conditions and minimum activities expected, dedicated time, compensation, reporting, teamwork and respect for differences, and a confidentiality agreement section to be signed (electronically) and kept by coordination. Selection should be carried out by the CAB coordinator him/herself (delegating this function has shown to mislead selection). The CAB membership conditions should be exposed and clarified, and ToR should be provided to candidate/member. Availability, gender balance and equity should be among minimum criteria. Level education is secondary to demonstrated interest to learn and deliver, and willingness to participate in policy discussion. Multi-profile selection is an asset for a CAB.
5. **CAB core functions** – core functions to any CAB are: (i) Analyse and provide input to the study protocol and research/program instruments, such as questionnaires, interview guides, training or informative materials; (ii) Organize and/or participate in regular meetings, capacity building sessions (training), seminars (webinars), conferences; (iii) To attend minimal number of planned activities (that may include reach out activities);

(iv) provide feedback to the stakeholders outside the study/project (informative sessions in forums, committees, councils); (v) helping disseminate study results. It is expected CAB members continue in their functions when advocating for the implementation of study results and policy discussion.

6. **Members evaluation** – Performance (quality) by members is quite subjective and dependent of several variables and intervenient and are context specific. Yet, regular, annual evaluation should take place in open discussions. Although activities can be numbered and counted, performance is to be evaluated (and self-evaluated), having qualitative tools as the reference. Non-performing members (regularly absent, or with disturbing attitudes) should be replaced for the sake of the group. Keeping non-performing or disruptive participants damages the entire CAB performance, as shown in other CAB experiences.
7. **CAB members motivation** – this item is crucial to keep a good performance by CAB members and require constant interaction with the coordination team (coordinator and assistant). Encouraging and acknowledging participation, providing background to their reports, including members in programs where they will have own expression is highly recommended.

Critical CAB add-on items

Here are points to be taken in consideration by the coordination team but also be shared and agreed with CAB members

i. Knowing or learning the stakeholders' scenario, being proactive and reaching out to health and program managers and other key actors in advance increases group performance.
ii. Keeping constant communication with PI and research team, but holding accountability to CAB members leadership, meaning the CAB coordinator leads CAB and avoid direct intervention by other research team members or managers, which will rapidly jeopardize the CAB as a cohesive group.
iii. Encouraging CAB members autonomy to voice their perception and expectations, and provide regular and open feedback and suggestions, besides supporting their own initiatives to design projects, studies will strengthen the group and encourage growth and learning.

References

1. Silva N de F, Oliveira PS de. Nada sobre Nós sem Nós: O papel do Comitê Comunitário de acompanhamento e assessoria em pesquisa na democratização dos estudos em Tuberculose: Nothing about us Without us: The Role of the Community Advisory Board in Democratizing Research and Tuberculosis. REPIS (Revista de Educação, Pesquisa e Informação em Saúde). 2025 Dec 3;e0478–e0478. doi:10.71209/repis.2025.4.e0478

2. Brasil. Ministério da Saúde. Secretaria de Vigilância em Saúde. Departamento de Vigilância das Doenças Transmissíveis., Secretaria de Vigilância em Saúde, Departamento de Vigilância das Doenças Transmissíveis. Técnicas de aplicação e leitura da prova tuberculínica [Internet]. 1a edição. Brasília: Ministério da Saúde; 2014 [cited 2022 Aug 27]. 56 p. Available from: https://bvsms.saude.gov.br/bvs/publicacoes/tecnicas_aplicacao_leitura_prova_tuberculinica.pdf

3. Cordeiro DC, Kuabara P, Amaral BC, Portugal LM, Sacramento DS, Rodrigues de Oliveira L, et al. Needs assessment and preparedness of the primary health care network for scaling-up preventive tuberculosis treatment in 5 Brazilian capitals. PLoS One. 2025;20(6):e0326428. doi:10.1371/journal.pone.0326428 PubMed PMID: 40577400; PubMed Central PMCID: PMC12204585.

4. Cordeiro DC, Aguiar EB de, Luiz RR, Sacramento DS, Paolino PLW, Gonçalves MJF, et al. Effect of successive tuberculin skin test readings on healthcare professionals' proficiency: an operational observational study in five high-tuberculosis-burden Brazilian municipalities, 2023-2024. Epidemiol Serv Saude. 2025;34:e20240795. doi:10.1590/S2237-96222025v34e20240795.en PubMed PMID: 41191681; PubMed Central PMCID: PMC12582531.

5. Gloria L de L, Bastos ML, Santos Júnior BD, Trajman A. A simple protocol for tuberculin skin test reading certification. Cad Saude Publica. 2021;37(8):e00027321. doi:10.1590/0102-311X00027321 PubMed PMID: 34495087.

6. NOTA INFORMATIVA Nº 14/2024-CGTM/.DATHI/SVSA/MS. 2024.
